# CMOS-Compatible Memristor for Optoelectronic Neuromorphic Computing

**DOI:** 10.1186/s11671-022-03744-x

**Published:** 2022-11-07

**Authors:** Facai Wu, Chien-Hung Chou, Tseung-Yuen Tseng

**Affiliations:** Institute of Electronics, National Yang Ming Chiao Tung University, Hsinchu, 30010 Taiwan

## Abstract

Optoelectronic memristor is a promising candidate for future light-controllable high-density storage and neuromorphic computing. In this work, light-tunable resistive switching (RS) characteristics are demonstrated in the CMOS process-compatible ITO/HfO_2_/TiO_2_/ITO optoelectronic memristor. The device shows an average of 79.24% transmittance under visible light. After electroforming, stable bipolar analog switching, data retention beyond 10^4^ s, and endurance of 10^6^ cycles are realized. An obvious current increase is observed under 405 nm wavelength light irradiation both in high and in low resistance states. The long-term potentiation of synaptic property can be achieved by both electrical and optical stimulation. Moreover, based on the optical potentiation and electrical depression of conductances, the simulated Hopfield neural network (HNN) is trained for learning the 10 × 10 pixels size image. The HNN can be successfully trained to recognize the input image with a training accuracy of 100% in 13 iterations. These results suggest that this optoelectronic memristor has a high potential for neuromorphic application.

## Introduction

Vast amounts of data storage and rapid information processing are desired nowadays [1, 2]. With the gradual failure of Moore’s law and the limitation of the von Neumann bottleneck, the revolutionary computing technique, neuromorphic computing is developed as the next-generation computing system due to its high-efficient information processing with low power consumption [3–5]. In a neuromorphic computing system, the synapses are crucial for connecting neurons and enabling the brain to function; an efficient artificial synapse is the core component [6, 7]. The two-terminal memristor is a promising candidate as an artificial synapse due to its compact synapse-like structure, low power consumption, high durability, easy integration, and unique nonlinear characteristic [8].

In general, most artificial neuromorphic computing systems are based on electrically excited memristors, which are limited by package density, parallel operation, and bandwidth [9, 10]. The operating speed of electronic memristors is limited by the trade-off between bandwidth and interconnection density. Compared with electrical tuning, optical control is a simple and low power consumption method to store and process data in an unprecedented bandwidth and high-speed optical way [11–14]. It can achieve programming by converting light information into an electric response [15].

However, there are remaining challenges, for example, process issues. Fully CMOS process-compatible optoelectronic memristors were rarely reported. In addition, most optoelectronic memristors show a nonvolatile light-induced current decrease phenomenon under visible light [16–19]. However, in this study, photonic current potentiation is realized under 405 nm light irradiation in the fully CMOS process-compatible ITO/HfO_2_/TiO_2_/ITO optoelectronic memristor. Neuromorphic computing is also investigated in this device by presenting an online learning pattern recognition.

## Device Fabrication and Characterization

**Fig. 1:**
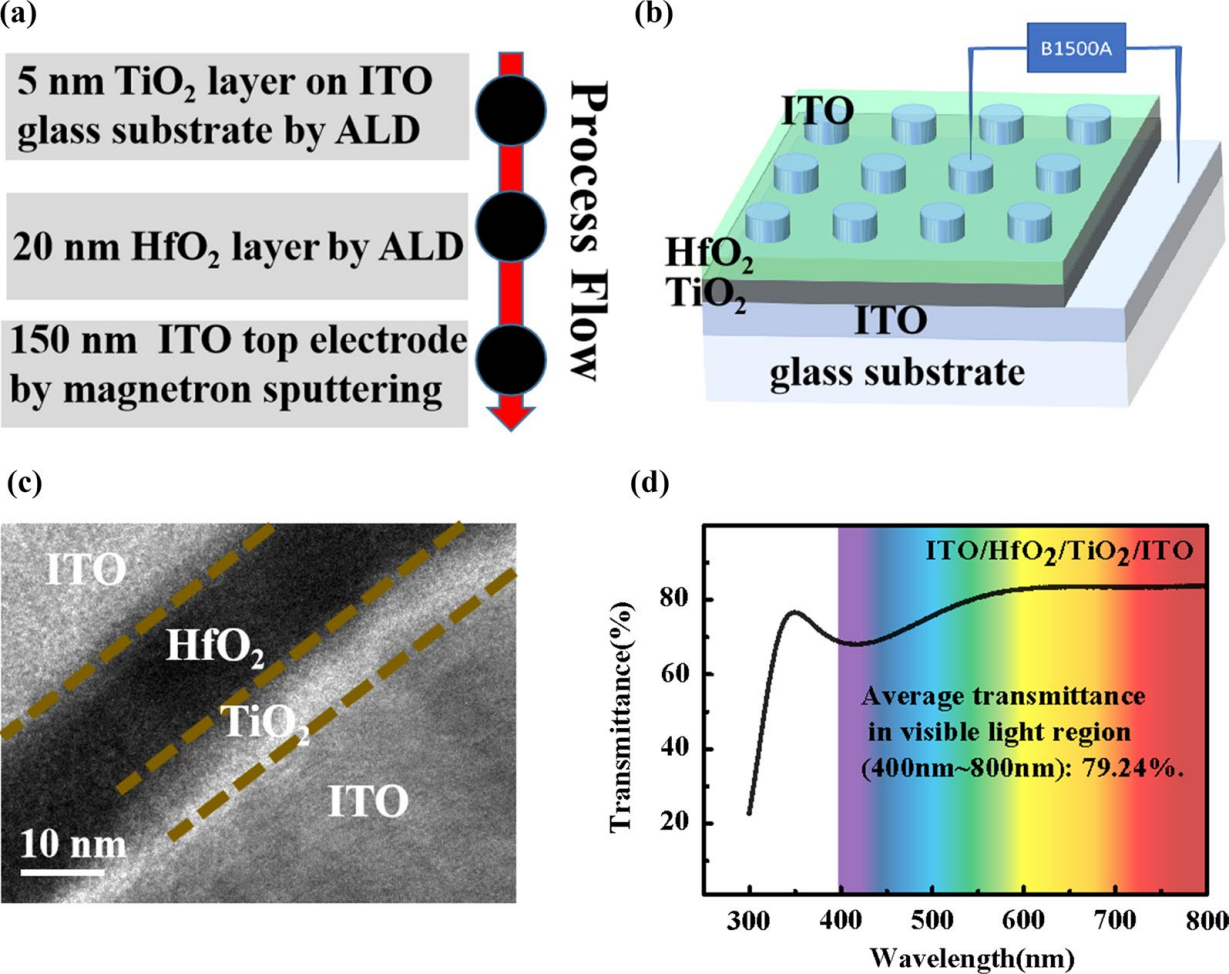
**a** Device fabrication process. **b** Schematic description of the ITO/HfO_2_/TiO_2_/ITO memristors. **c** Typical cross-sectional TEM image of the device. **d** UV–visible spectrum of the device

The ITO/HfO_2_/TiO_2_/ITO optoelectronic memristor was prepared, and the process flowchart is shown in Fig. 1a: Firstly, a 5-nm TiO_2_ interface layer was deposited on the bottom electrode (BE) ITO-coated glass substrate by atomic layer deposition (ALD). Then, a 20-nm HfO_2_ switching layer was grown by ALD. Finally, magnetron sputtering was used to deposit 150-nm ITO top electrodes (TEs) with a hard mask. The schematic structure of the device is depicted in Fig. 1b. The electrical characteristics of the device were measured with the semiconductor parameter analyzer (Agilent B1500). During the electrical test, the voltage was applied to the TE while BE was grounded. The cross-sectional high-resolution TEM (HRTEM) image of the device (Fig. 1c) indicates that the boundaries between each layer are clear and the film quality of each layer is good. The transparency of the device was measured by UV–visible spectroscopy, and the result is shown in Fig. 1d. An average transmittance of more than 79.24% in the visible region is achieved in this memristor, which demonstrates its high transparency and potential for application in photoelectric neuromorphic computing system.

## Results and Discussion

**Fig. 2:**
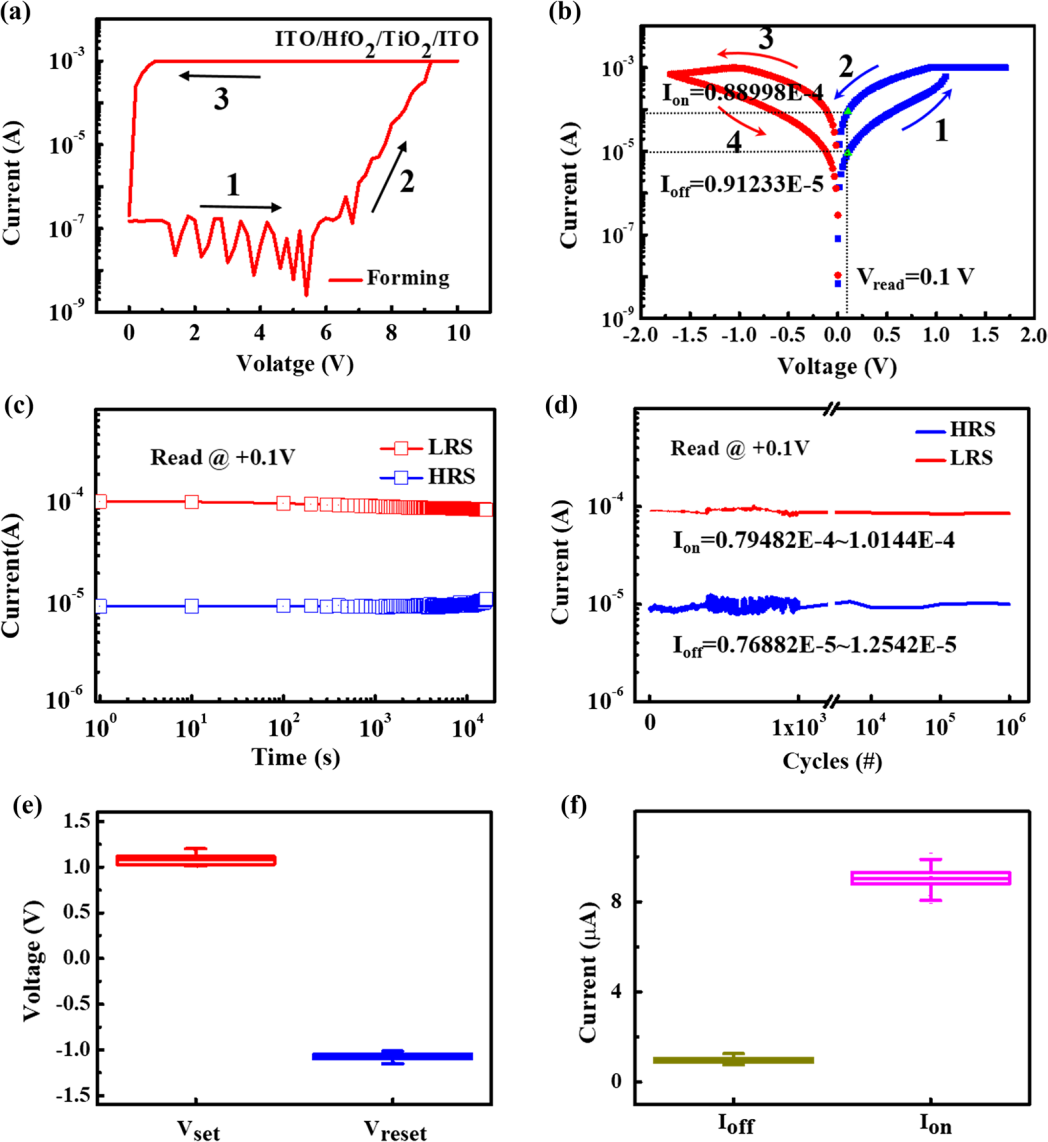
**a** Electroforming process of the device. **b** I–V curve. **c** Retention of the device at room temperature. **d** Endurance plot. **e** Set and reset voltage distributions. **f** On and off current distributions

The RS characteristics of the device are depicted in Fig. 2. An electroforming operation is required for the fresh device to initialize the subsequent RS behavior. When a positive sweeping voltage (0 → 10 V) with 1 mA compliance current (*I*_CC_) is applied during the electroforming process, the current gradually increases at about 6 V and reaches *I*_CC_; thus, the device turns to the low resistance state (LRS), as shown Fig. 2a. The electroforming voltage is a little high and can be decreased or even forming-free by decreasing the deposition film thickness [20] of HfO_2_ layer or using metal doping [21, 22]. After electroforming, under a negative sweeping voltage (0 →  − 1.7 V), namely RESET process, the current gradually decreases, demonstrating that the device turns from LRS to a high resistance state (HRS). Then, under the SET (0 → 1.7 V) and RESET (0 →  − 1.7 V) processes, the device can switch repeatedly between LRS and HRS with 1 mA *I*_CC_, as shown in Fig. 2b. Both SET and RESET processes are analog switching, which is beneficial for neuromorphic computing [23]. The electrical switching phenomenon can be attributed to the formation of oxygen vacancies (V_O_^2+^) conductive filaments during electric stimulation [23]; such a switching mechanism is widely accepted for explaining the conduction phenomenon of the memristors. The big difference between forming voltage and set voltage can be explained as follows: During the forming process, a positive voltage is applied to the ITO top electrode (TE), the oxygen ions (O^2−^) move toward TE and store in the TE, and the Vo^2+^-based conductive filament would be formed at HfO_2_/TiO_2_ resistive layer and grow up to connect TE and bottom electrode (BE). The device turns to a low resistance state (LRS). This process needs a high voltage to cause a soft breakdown and generate the point defect of Vo^2+^ due to the high resistance of the pristine device. During the reset process, a negative voltage is applied to the TE, and the O^2−^ ions move from the TE to the BE. The O^2−^ would combine with the Vo^2+^ in the resistive layer to disrupt the conductive filament. The device is changed to a high resistance state (HRS). It is worth noting that only a part of the conductive filament, which exists at the near HfO_2_/TiO_2_ interface, would be broken to achieve HRS [23]. Therefore, during the next set process, it only needs a much lower voltage to fix this part of the filament to provide LRS.

The retention characteristics of the device on LRS and HRS states are investigated, as shown in Fig. 2c. The resistance values of both states maintain stability and show no obvious shift beyond 10^4^ s. The endurance performance was also studied, and the result is indicated in Fig. 2d. The endurance test shows that the switching characteristic of the device does not have any degradation with 10^6^ switching cycles. The cycle-to-cycle variability of *V*_set_, *V*_reset_, *I*_off_, and *I*_on_ is shown in Fig. 2e, f, respectively. *V*_set_ and *V*_reset_ are extracted from 100 switching cycles, while *I*_off_ and *I*_on_ are extracted from 1000 switching cycles. These results show the extremely narrow distribution of operating voltage and current, meaning that the device shows excellent cycle-to-cycle uniformity. With low variability, the conductance of the memristor will be programmed precisely in the neural network, and calculation and iteration will be more efficient, which can achieve high accuracy and need fewer train epochs to compute.

**Fig. 3:**
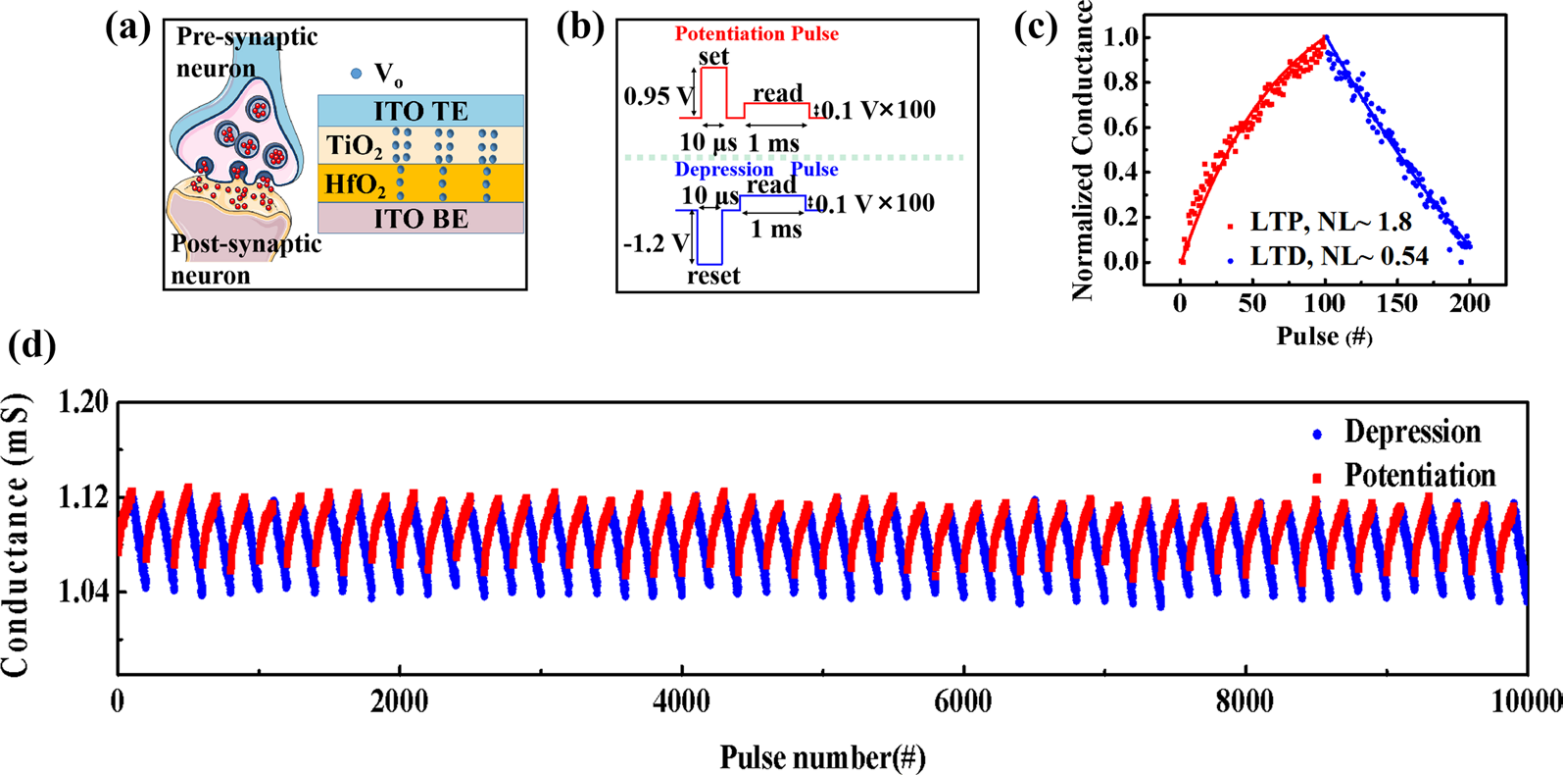
**a** Schematic diagram of the synapse and the structure of the ITO/HfO_2_/TiO_2_/ITO synaptic device. **b** Pulsing schemes for potentiation and depression, respectively. **c** Gradual conductance modulation of potentiation and depression under successive pulse stimulation. **d** Stable 50 epoch potentiation and depression trainings

Biological synapses are the information transmission centers between pre-neurons and post-neurons, and the transmission process is completed by the transmission of neurotransmitters, which are between the presynaptic membrane and the postsynaptic membrane [24]. The spike potential or action potential of presynaptic neurons can be transmitted through synapses to generate postsynaptic potentials. The amplitude of postsynaptic potentials depends on the weight of the synapse [25]. Adjustable resistance allows the memristor to mimic the typical synaptic response of the brain [26]. The schematic diagram of the synapse and the structure of the device are shown in Fig. 3a. After the above electrical test, the device was used for mimicking long-term potentiation (LTP) and depression (LTD) synaptic behaviors. As shown in Fig. 3b, set pulses (+ 0.95 V, 10 μs) are applied for potentiation and reset pulses (− 1.2 V, 10 μs) are employed for depression, with a reading pulse (0.1 V, 1 ms). After repeating the set pulse scheme 100 times, the conductance increases gradually, 1.8 coefficient of nonlinearity (NL) potentiation is realized. Then, following the 100 times reset pulse scheme, the conductance decreases gradually and 0.54 coefficient of NL depression is revealed, as shown in Fig. 3c. In addition, the device can be trained more than 50 stable epochs without degrading the dynamic range (1.04–1.12 mS), as shown in Fig. 3d. These results indicate the potential of this memristor for neural network applications [27, 28].

**Fig. 4:**
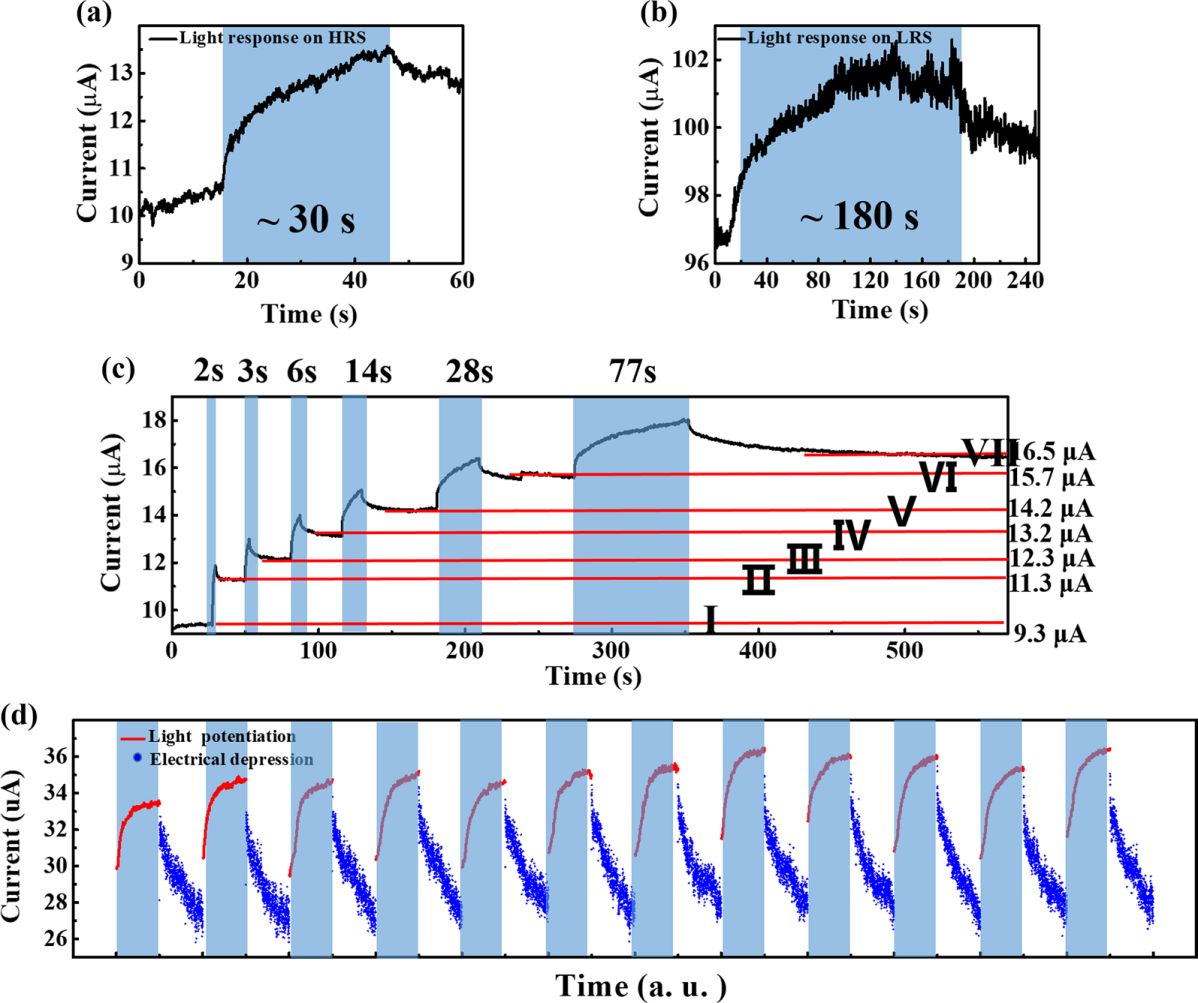
Current response under the application of 405 nm wavelength light irradiation on **a** HRS and **b** LRS. **c** Multilevel storage realized by light irradiation. **d** Optical potentiation and electrical depression characteristics simulated by optoelectronic artificial synapse

Optoelectronic memristor has opened up one way for light-tunable synaptic weight to further transmit and process stimulus information [29]. The light-tunable synaptic activities are investigated as follows. As shown in Fig. 4a, the initial current is about 10.5 μA on HRS. When a 405 nm, 100 mW/cm^2^ light pulse is applied from 16 to 46 s, light information could be perceived and the current gradually increases to 14 μA. On LRS, the current also increases from 97 to 102 μA with 180 s light irradiation, as shown in Fig. 4b. The transition time and switching energy efficiency under illumination are not good enough, but they can be improved by doping modification [30–34]. The photoresponse current stems from the light irradiation-induced oxygen vacancies (Vo^2+^), which will be discussed later. For both LRS and HRS, with stronger optical pulse intensity, more e-/Vo^2+^ pairs will be generated, and the accumulated Vo^2+^ can form conductive filaments to increase the conductance. The difference between LRS and HRS during illumination is that the current amplification on LRS is less than that on HRS since there were already existing many Vo^2+^ on LRS before illumination. The amount of light-induced Vo^2+^ is relatively fewer compared with already existing Vo^2+^; thus, the increase in current on LRS is less than that on HRS. With the same initial current level and illumination time/rise time, stronger optical pulse intensity will induce a higher maximum/final potentiation current. In other words, with the same initial current level and stronger optical pulse intensity, it needs a shorter illumination time/rise time to achieve the same maximum/final potentiation current. After removing the light, the maximum current will decay to the final current during the falling time, which is related to the spontaneous physical diffusion of Vo^2+^ conductive filaments component into the switching layer, driven by interfacial-energy-related Gibbs–Thomson effect [35] and Rayleigh instability of nanosize CFs [36]. To minimize the interfacial energy, the filaments component slowly diffuses to the minimum energy positions and merges into larger clusters. The driving force for this process is the chemical potential gradient induced by a perturbation in the radius. The instability can be modeled by introducing a sinusoidal perturbation with a form $r = r_{0} + \delta \sin (2\pi z/\lambda )$ on the surface of cylindrical CF, where $r_{0}$ is the initial CF’s radius, $\delta$ and $\lambda$ are the amplitude and wavelength of the perturbation, respectively, and *z* is the coordinate along the CF’s axis. The cylindrical CF will become unstable when $\lambda > 2\pi r_{0}$. At a certain wavelength $\lambda_{m} = 2\sqrt 2 \pi r_{0}$, there is a minimum characteristic time of perturbation $(\tau_{m} )$, which corresponds to the CF’s relaxation time from the initial cylinder to its final shape [36]. The multilevel storage capacity of a memristor under light irradiation is vital for a light-in-memory computing system, and the related result of the investigation is shown in Fig. 4c. The current increases under various times of duration (2, 3, 6, 14, 28, 77 s). After removing the light, the current will not return to the initial state immediately but remains at a higher level. During the decay process/fall time, the current obeys this formula:

1 $$I = Ae^{( - t/\tau )} + I_{{{\text{final}}}}$$

where *A* is the part of the unstable state current, which will be dissipated after a period of time when the light is off. The value of *A* depends on the light intensity; *A* will increase with stronger light intensity. $I_{{{\text{final}}}}$ is the final state current after removing the light, which depends on the filament morphology. $A + I_{{{\text{final}}}}$ means the initial current on the moment when the light is off. $\tau$ is the average time it takes for unstable state current to be dissipated, which means the time at which the $e^{ - t/\tau }$ is reduced to 1/*e*. $\tau$ is related to the diffusion kinetics. Take the decay process from 350 to 545 s (*t* = 350–545) as an example; $A$, $\tau$, and $I_{{{\text{final}}}}$ are fitted as 9.63123E−4 A, 51.32848 s, and 1.65104E−5 A, respectively. As shown in Fig. 4c, 7 stable states are realized after different illumination times, indicating that the memristor has the capability of in situ optical sensing and storage. By designing successive light and electrical pulses, the potentiation and depression behaviors of artificial synapses can be simulated, as shown in Fig. 4d. The current of the memristor increases during light irradiation (405 nm, 100 s) and decreases during the electrical pulse (− 1.7 V, 10 μs, 1000 times), corresponding to the LTP/LTD characteristics of a dynamic range of 26–36 μA. These results show that this memristor can simulate basic synaptic functions under external light signals.

**Fig. 5:**
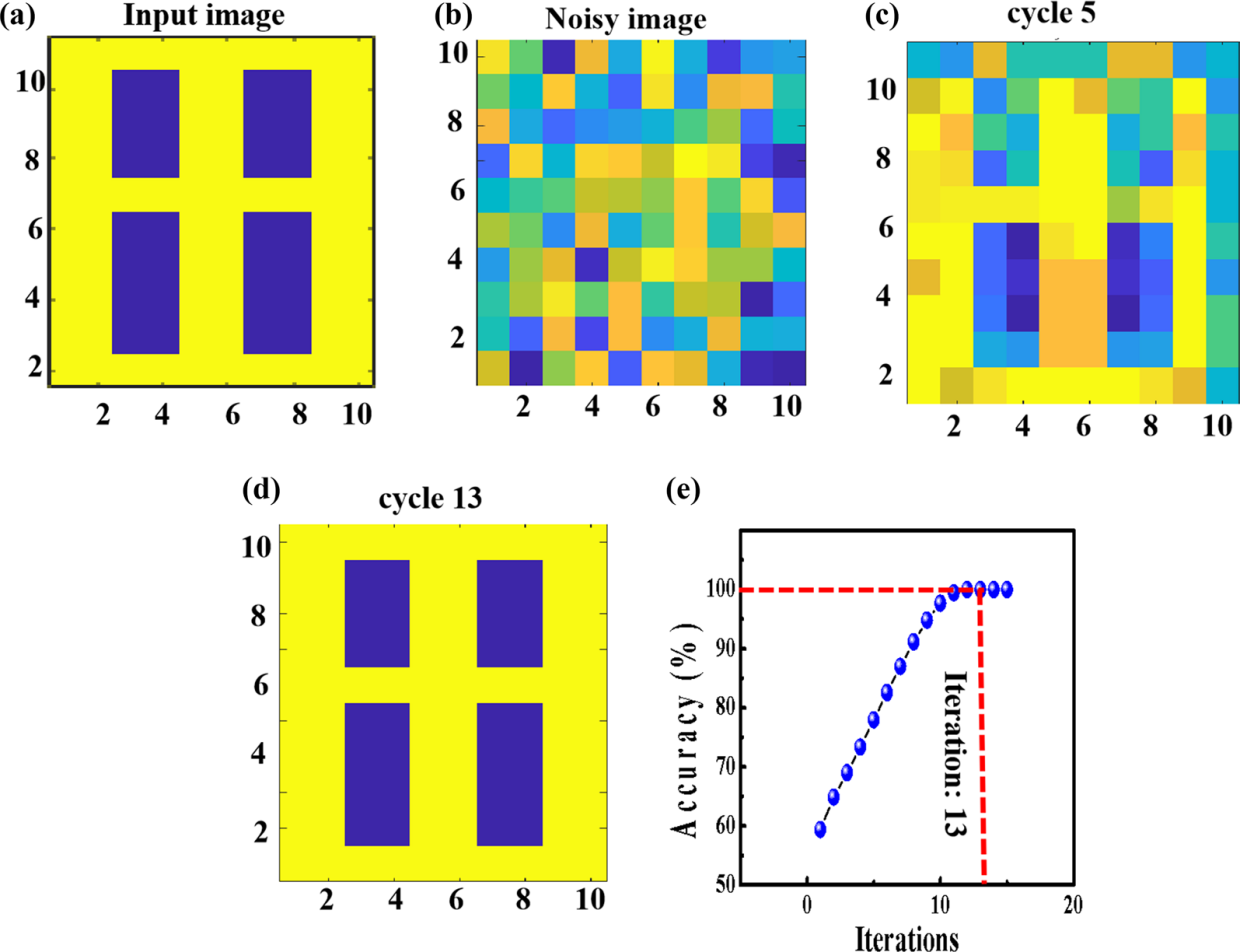
Pattern recognition simulation. **a** Input image of 10 × 10 pixels size for recognition. **b** Noisy image after weight updating. **c** Recalled image after 5 cycles of iteration. **d** Recalled image after 13 cycles of iteration. **e** The evolution of accuracy versus number of iterations

Information processing, such as learning, is vital to biological systems [37]. Tunable memristor conductance can simulate continuous modulated synapse weight to achieve efficient neuromorphic calculation and recognition functions [38]. Based on the optical potentiation and electrical depression of conductances, we employed the Hopfield neural network (HNN) to investigate the pattern recognition capability of the device; the HNN is a form of recurrent ANN (Hopfield, 1982, and Little, 1974) [39]. The simulated Hopfield neural network (HNN) is trained to learn the 10 × 10 pixels size image, as shown in Fig. 5a. We used relative normalized memristor conductance of optical potentiation and electrical depression to carry out the weight map simulation; each pixel represents the conductance of a single synapse. Initially, each synapse is randomized to store information in the range between 0 (yellow color) and 1 (blue color) (Fig. 5b) to form the noisy image. Then, the value of each pixel will be updated during the learning process. The outcomes of the images after 5 and 13 cycles are depicted in Fig. 5c, d, respectively. The HNN can be successfully trained to identify the input image in 13 cycles of iteration (Fig. 5e) with 100% accuracy. In general, the results show that the ITO/HfO_2_/TiO_2_/ITO device can be possibly used for neuromorphic applications.

**Fig. 6:**
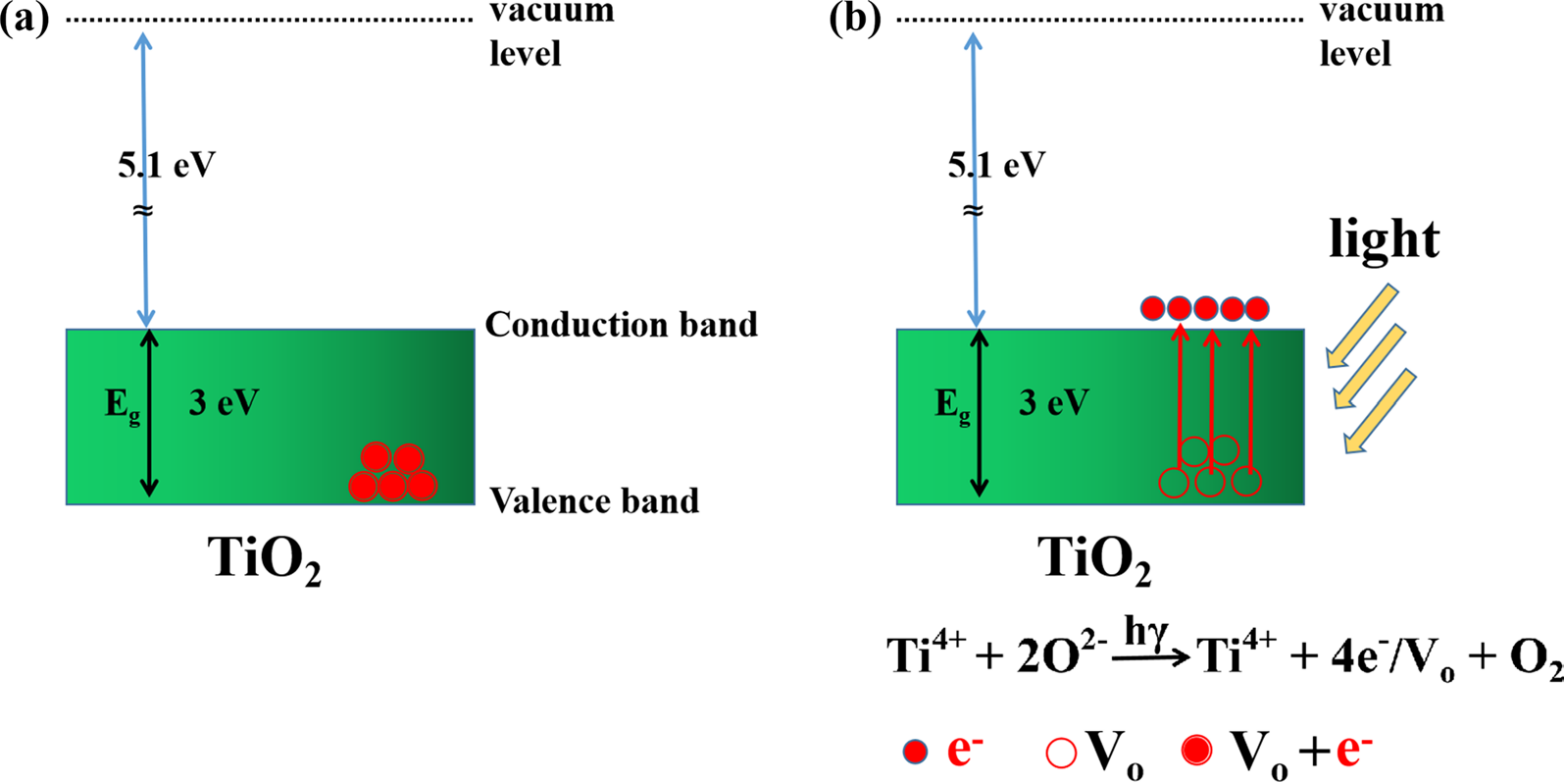
Light-tunable mechanism of the device. **a** Energy band diagram of TiO_2_ film. **b** Formation of V_O_^2+^ in TiO_2_ film under the illumination of light

A detailed comparison between previously reported photoelectronic artificial memristor synapses and the present device is provided in Table 1. On the whole, the comparison indicates that the performance of the present device is better than those of the previously reported synaptic devices. The light-tunable mechanism could be explained by the light irradiation-induced oxygen vacancies (Vo^2+^) in the TiO_2_ layer [30], which form conductive filaments to increase the conductance. As shown in Fig. 6a, the energy band gap of TiO_2_ is 3 eV, and the energy of 405 nm light is 3.0612 eV, which is higher than the TiO_2_ energy band gap. Under 405 nm light irradiation, the electrons of neutral lattice oxygen will gain energy *hγ*, activated to the conduction band. This action leaves movable O^2−^ (counterpart Vo^2+^) in TiO_2_ film as shown in Fig. 6b. The oxygen ions are combined into oxygen gas, contributing to generating more Vo^2+^. With continuous irradiation, Vo^2+^ would accumulate in the TiO_2_ layer. With enough amount of Vo^2+^, they will aggregate and form conductive filaments, thus realizing an optical conductance increase. The HfO_2_ layer has no current response under 405 nm light irradiation since the energy band gap of HfO_2_ is 5.7 eV, much higher than photon energy.

**Table 1:** Comparison between previous artificial photoelectronic memristive synapses and this work

Memristor	# of conductance	Endurance	Retention time	Light source	In-sensor computing	COMS compatible	Refs.
Ag/ZnO/ITO	25	500	10^4^ s	Visible light	Yes	No	[40]
W/MoS_2_/p-Si	20	15	150 s	Ultraviolet	Yes	No	[41]
ITO/Nb:SrTiO_3_/Ag	100	NA	3 × 10^3^ s	Visible light	Yes	No	[42]
Al/TiN_x_O_2−x_/MoS_2_/ITO	400	450	30 s	Visible light	Yes	No	[43]
ITO/ZnO_1−x_/AlO_y_/Al	30	1000	500 s	Ultraviolet	Yes	No	[44]
ITO/HfAlO/TiN-NP/HfAlO/ITO	100	175	NA	Ultraviolet	Yes	No	[45]
Al/TiS_3_/ITO	50	100	10^4^ s	Visible light	Yes	No	[46]
ITO/HfO_2_/TiO_2_/ITO	100	1000	16,300	Visible light	Yes	Yes	This work

## Conclusions

In summary, the fully CMOS process-compatible ITO/HfO_2_/TiO_2_/ITO optoelectronic synaptic memristor was fabricated. High transmittance under visible light was realized to ensure photosensitization. Stable bipolar analog switching, beyond 10^4^ s data retention, and endurance of 10^6^ cycles were achieved as basic storage function. Synaptic functions including LTP, LTD, and photonic potentiation were established. The light-tunable behavior originates from light irradiation-induced Vo^2+^. Furthermore, after 13 cycles of iteration, the simulated HNN can successfully recognize the 10 × 10 pixels size image. This memristor shows great potential in the next generation of intelligent optoelectronic neuromorphic computing systems.

## Data Availability

The data that support the findings of this study are available from the corresponding author upon reasonable request.
